# Trauma, beauty and the divided self: what Dostoevsky offers contemporary psychiatry

**DOI:** 10.1192/bjo.2026.11026

**Published:** 2026-04-21

**Authors:** Hala Kerbage, Philippe Courtet

**Affiliations:** Department of Child and Adolescent Psychiatry, Saint-Eloi Hospital, University Hospital of Montpellier, Montpellier, France; Center for Epidemiology and Population Health, INSERM, Paris-Saclay University, Paris, France; Department of Emergency Psychiatry and Acute Care, Lapeyronie Hospital, University Hospital of Montpellier, Montpellier, France; IGF, University of Montpellier, CNRS, INSERM, Montpellier, France

**Keywords:** Dostoevsky, trauma, dissociation, beauty, literature

## Abstract

This commentary reflects on what Dostoevsky’s work offers contemporary psychiatry, showing how his portrayals of fractured selves, dissociation, shame and the ambivalent force of beauty deepen our understanding of trauma’s lived experience and highlight the psychological complexities that clinical language cannot always capture.

**Figure:**
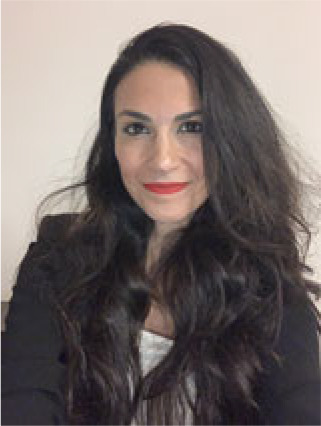

Few writers have penetrated the human soul with as much psychological intensity as Fyodor Dostoevsky. His novels, marked by personal catastrophe and profound spiritual inquiry, read like case notes written long before psychiatry had a name for the states he described. Dostoevsky lived through trauma that reshaped his inner world – a mock execution, years in Siberian labour camps, chronic illness – and his work carries the imprint of that wound. For clinicians today, his writing offers a rich, unsettling map of trauma’s effects on consciousness, agency, desire and the fragile movement towards beauty and meaning. More broadly, literature has long offered a unique access to experiences of psychological distress that escape psychiatric categorisation.^1^ Through narrative and metaphor, it captures states such as shame, fragmentation, moral conflict and despair within their social and relational contexts, without seeking to resolve their ambiguity. Dostoevsky’s literary universe stands within this tradition while bringing it to an exceptional degree of psychological and moral intensity.

His writing has long attracted sustained attention from psychiatrists, neurologists and literary scholars, particularly regarding epilepsy, representations of altered consciousness and depictions of psychological suffering.^2–7^ The present commentary engages with this body of work from a trauma-informed, phenomenological perspective, highlighting how these narratives continue to illuminate dimensions of subjective traumatic experience that remain clinically relevant today.

Dostoevsky’s insight begins with his own experience. In 1849, he stood in front of a firing squad, certain he had minutes left to live, before being reprieved at the last moment. What he later described was a collapse of temporal structure: time thickened, sensations sharpened, life appeared simultaneously hyperreal and unreal. Trauma research today refers to such experiences as dissociation – a disruption of the continuity of experience that reorganises memory, embodiment and identity.^8^ Experiences of dissociation in Dostoevsky’s work may reflect overlapping contributions from both traumatic exposure and neurological vulnerability. In addition to his experience of extreme trauma, Dostoevsky lived with long-standing epilepsy, a diagnosis supported by converging historical and medical accounts.^2^ Contemporary research highlights phenomenological overlaps between epilepsy – particularly temporal lobe epilepsy – and dissociative experiences, including altered states of consciousness, depersonalisation and disruptions of self-experience.^9^ While Dostoevsky’s literary insights cannot be reduced to a neurological condition, the coexistence of trauma and epilepsy likely sharpened his sensitivity to the fragility and discontinuity of consciousness that permeate his portrayals of the divided self. Across his characters, this sensibility takes the form of recurring configurations of psychic rupture that resonate with contemporary understandings of trauma, dissociation and stigma, grounded in a shared experiential core: the fragmentation of the self under moral, relational and existential strain.

Prince Myshkin in *The Idiot* is perhaps the most striking illustration of Dostoevsky’s portrayal of vulnerability at the intersection of trauma and epilepsy. His extreme openness and emotional permeability render him exquisitely sensitive to others’ suffering yet leave him without adequate psychic protection. His encounters with Nastasya Filippovna reveal trauma’s corrosive effects on identity and self-worth. Marked by humiliation and shame, she oscillates between a longing for redemption and a compulsive pull towards self-destruction. Within the figure of Prince Myshkin, and in the relational space that unfolds between him and Nastasya Filippovna, Dostoevsky anticipates what contemporary psychiatry has described as ‘double stigma’, whereby neurological illness and mental suffering intersect to compound experiences of shame and social exclusion.^10^ Their relationship exposes how complex trauma shapes attachment, drawing individuals into cycles of idealisation, rejection and despair.^11^ Here, dissociation appears less as detachment than as instability – an inability to integrate tenderness, desire and self-respect into a coherent sense of self. The figure of Prince Myshkin is inseparable from Dostoevsky’s own lived experience of epilepsy, which profoundly shaped his understanding of altered consciousness, vulnerability and affective intensity.^2,4^ This oscillation between lucidity, emotional openness and collapse resonates with both trauma-related dissociation and peri-ictal and inter-ictal experiential states described in epilepsy.

In *Crime and Punishment*, Raskolnikov’s crime unfolds in a state of psychic narrowing that resembles a dissociative fugue. His actions feel both deliberate and unreal, as though carried out by a self partially detached from its own agency. In the aftermath, guilt does not function as a discrete emotion but as an atmosphere that permeates perception, bodily experience and thought. His oscillation between emotional numbing and intrusive moral anguish evokes what contemporary clinicians might recognise as moral injury – a collapse of self-coherence following an irreconcilable violation of deeply held values.^12^ Rather than moving towards resolution, Raskolnikov remains suspended between grandiosity and self-annihilation, illustrating how trauma fractures continuity of identity.

These patterns reach a more explicit philosophical articulation in *The Brothers Karamazov*. Ivan Karamazov’s hallucinatory dialogues with the devil give narrative form to the disintegration of thought under unbearable moral tension. His suffering is not reducible to madness, but reflects the psychic cost of confronting cruelty, injustice and responsibility without the possibility of symbolic resolution. In a different register, figures such as Stavrogin move through the world with a deadened affect and a profound disconnection from their inner life, anticipating what psychiatry now describes as structural dissociation, in which parts of the self are split off to survive overwhelming experience.^8^

In Dostoevsky’s universe, trauma is inseparable from beauty. For him, beauty is an inner force, a striving towards coherence, dignity and transcendence. Beauty awakens longing, disrupts dissociation and brings the self closer to its wounds. It is capable of saving, but also of tearing open. This duality resonates with trauma-informed therapy, where moments of meaning, intimacy or tenderness can catalyse healing but also trigger buried pain. The often quoted line from *The Idiot* ‘beauty will save the world’ should not be read as simple idealism. In Dostoevsky’s novels, beauty saves only by demanding transformation. For trauma survivors, reclaiming beauty, whether aesthetic, relational or spiritual, is often part of reconstructing a sense of self after devastation. But Dostoevsky also warns that ideals can become tyrannical, especially when used to flee shame or emulate unattainable perfection. Many of his characters are tormented by ideals that both inspire them and crush them – an experience clinicians often see in patients whose internalised demands for purity, moral perfection or self-sacrifice deepen their suffering.

What Dostoevsky ultimately offers psychiatry is a phenomenology of the divided self. Trauma in his work is a force that rearranges the architecture of the psyche. His characters live within contradictions, between guilt and pride, tenderness and violence, longing for connection and fear of intimacy. Rather than resolving these conflicts, he allows them to coexist, to generate movement. This mirrors modern understandings of recovery as learning to live with complexity, ambivalence and the slow reconstruction of agency.

Dostoevsky reminds us that trauma is not just a diagnostic entity but a lived experience – sensory, moral, relational, existential. His writing sharpens our empathy for what remains hidden or unspeakable in our patients and invites us to approach the psyche with both precision and imagination. The aim, however, is not to reduce Dostoevsky’s art to a psychopathological framing. Literature is powerful precisely because it resists categorisation, capturing the ambiguity and fragmentation that clinical language often flattens. By revealing these subjective textures, it helps us grasp the inner life beneath symptoms and exposes the limits of our diagnostic frames. For medical students, psychologists and psychiatry residents, engagement with literature can complement clinical training by fostering experiential understanding, narrative sensitivity and ethical reflection. Literary representations of distress help cultivate empathy^13^ and tolerance for ambiguity – core competencies for working with trauma and complex mental suffering that cannot be fully acquired through diagnostic reasoning alone. As humanities regain their place in medical education^14^ Dostoevsky’s literary art enriches psychiatry by returning us to the deep sources of psychological life, where suffering and beauty intertwine in the search for meaning.

## Data Availability

No new data were generated or analysed in support of this research.
